# Supplementary material for: The Construction and Exploration of a Comprehensive MicroRNA Centered Regulatory Network in Foxtail Millet (Setaria italica L.)
Source: Front Plant Sci. 2022 May 6;13:848474. doi: 10.3389/fpls.2022.848474 (PMC9121102; doi:10.3389/fpls.2022.848474)
Supplement: Supplementary file 2 [file Data_Sheet_2.zip › Sit-miR169e_Seita.9G066900.1_230_TPlot.pdf]

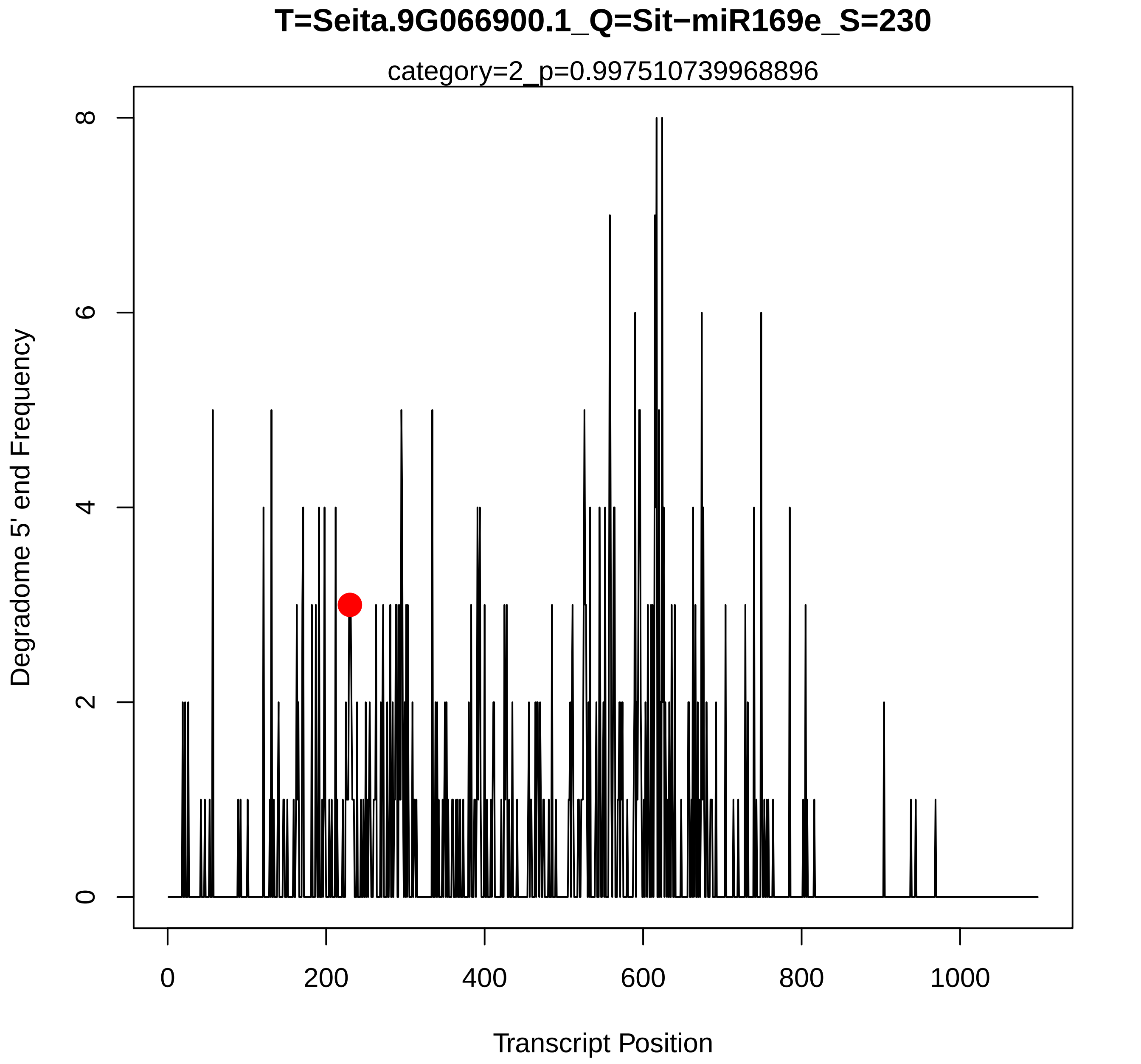
T=Seita.9G066900.1_Q=Sit−miR169e_S=230
category=2_p=0.997510739968896
Degradome 5' end Frequency
Transcript Position
0
2
4
6
8
0
200
400
600
800
1000
